# Supplementary material for: Continuous HIV-1 Escape from Autologous Neutralization and Development of Cross-Reactive Antibody Responses Characterizes Slow Disease Progression of Children
Source: Vaccines (Basel). 2021 Mar 14;9(3):260. doi: 10.3390/vaccines9030260 (PMC7999787; doi:10.3390/vaccines9030260)
Supplement: Supplementary file 1 [file vaccines-09-00260-s001.pdf]

Supplementary Material to

"Continuous HIV-1 escape from autologous neutralization and development of cross-reactive antibody responses characterizes slow disease progression of children."

by Stefania DISPINSERI et al.

Figure S1
Tables S1 and S2

Supplementary Table S1: List of Immunological assays performed with the plasma of each child.

Child code	Autologous neutralization (PBMC assay)	Heterologous neutralization (PBMC assay)	Heterologous neutralization (Tzmbl assay)	ADCC BaL gp120
Slow Progressor				
B193	x	x		x
B196	x			
B201	x			
B204	x	x	x	x
B224	x	x	x	x
B341			x	
B380	x		x	x
B256	x	x	x	x
B397			x	
Rapid Progressor				
B32	x	x	x	x
B199	x	x	x	x
B3	x		x	x
B115	x	x	x	x
B136	x	x	x	x
B145	x	x	x	x
B266			x	
B390		x	x	
B385			x	
B190	x		x	x
B271			x	
B306	x		x	x
B312			x	
B336			x	
B344			x	
B355			x	

Supplemental Table S2. Autologous neutralization of rapid and slow progressors against the contemporaneous virus and follow-up virus isolated.

Child	Virus [(a)]	Neutralization Contemporaneous	Follow-up [(b)]
		Rapid Progressor	
B201	3	0	-
	6	0	nd
B193	4	0	-
	21	0	nd
B196	1	0	-
	34	0	nd
B224	1	0	+
	3	40	+
	6	40	+
	18	0	+
	45	0	nd
B204	0	0	-
	1	0	-
	2	0	-
	38	0	-
B380	6	20	+
	19	0	+
	26	20	+
	34	20	+
	42	20	nd
B256	3	0	-
		Slow Progressor	
B199	3	0	+
	7	0	-
	37	0	-
B32	8	0	+
	42	320	+
	54	0	nd
B145	1	0	+
	5	0	+
	48	0	-
B3	6	20	+
	18	0	+
	54	0	+
B115	3	0	+
B136	3	0	+
	34	0	-
	60	0	-
	64	0	nd
B306	9	0	+
	16	20	+
	40	0	+
	52	0	+
	67	0	nd
B190	1	0	+
	11	0	+

Footnote to Supplementary Table 2: Neutralization titers, determined in a PBMC-based assay, are the reciprocal of the highest dilution giving a reduction of 90% of HIV-1 p24 antigen as determined in ELISA. 0 means dilution < 1/20. (a) Age in months at the moment of viral isolation; (b) "-" means that the viral isolate was not neutralized by sera collected after the viral isolation date; "nd" means that follow up sera to that virus was not available; "+" means neutralization of the viral isolates at following time point of sera.

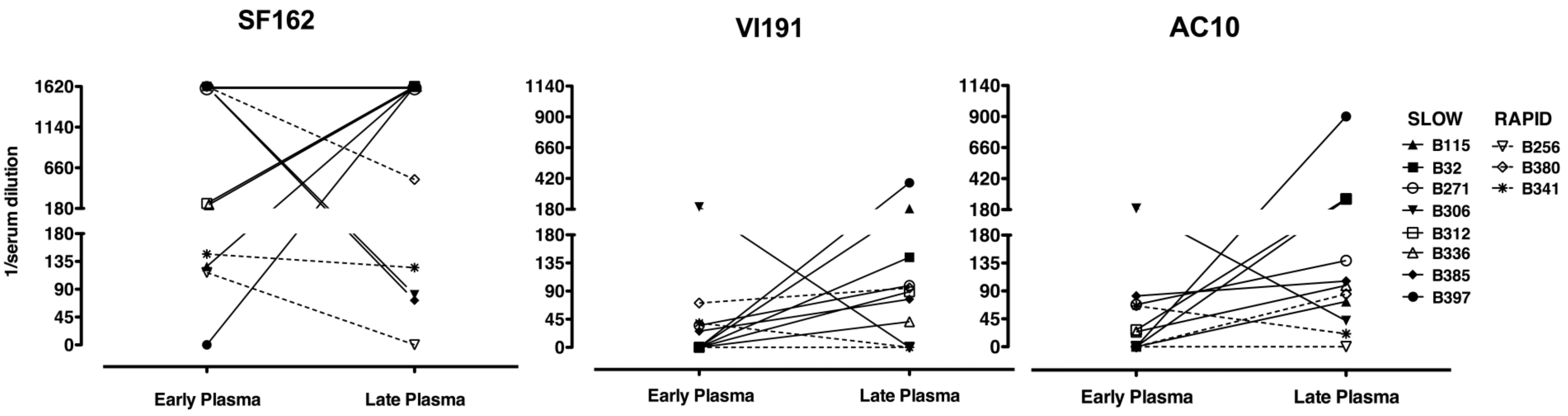

Supplementary Figure 1. Dynamics of neutralizing antibody response against heterologous PSVs tested in paired plasma samples from HIV-1 infected children. Indicated is the plasma dilution at which 50% inhibition was achieved with TZMbl-assay. The plasma dilution of 1/1620 was indicated when an end-point titer was not achieved within the last plasma dilution of the test. 0 means less than 1/20 plasma dilution.
